# Sex‐biased oviposition by a nursery pollinator on a gynodioecious host plant: Implications for breeding system evolution and evolution of mutualism

**DOI:** 10.1002/ece3.3014

**Published:** 2017-05-23

**Authors:** Laura A. D. Doubleday, Lynn S. Adler

**Affiliations:** ^1^ Graduate Program in Organismic and Evolutionary BiologyUniversity of Massachusetts AmherstAmherstMAUSA; ^2^ Graduate Program in EntomologyUniversity of Massachusetts AmherstAmherstMAUSA; ^3^ Biology DepartmentUniversity of Massachusetts AmherstAmherstMAUSA

**Keywords:** gynodioecy, *Hadena ectypa*, nursery pollination, plant breeding systems, sex‐biased interactions, *Silene vulgaris*

## Abstract

Dioecy, a breeding system where individual plants are exclusively male or female, has evolved repeatedly. Extensive theory describes when dioecy should arise from hermaphroditism, frequently through gynodioecy, where females and hermaphrodites coexist, and when gynodioecy should be stable. Both pollinators and herbivores often prefer the pollen‐bearing sex, with sex‐specific fitness effects that can affect breeding system evolution. Nursery pollination, where adult insects pollinate flowers but their larvae feed on plant reproductive tissues, is a model for understanding mutualism evolution but could also yield insights into plant breeding system evolution. We studied a recently established nursery pollination interaction between native *Hadena ectypa* moths and introduced gynodioecious *Silene vulgaris* plants in North America to assess whether oviposition was biased toward females or hermaphrodites, which traits were associated with oviposition, and the effect of oviposition on host plant fitness. Oviposition was hermaphrodite‐biased and associated with deeper flowers and more stems. Sexual dimorphism in flower depth, a trait also associated with oviposition on the native host plant (*Silene stellata*), explained the hermaphrodite bias. Egg‐receiving plants experienced more fruit predation than plants that received no eggs, but relatively few fruits were lost, and egg receipt did not significantly alter total fruit production at the plant level. Oviposition did not enhance pollination; egg‐receiving flowers usually failed to expand and produce seeds. Together, our results suggest that *H. ectypa* oviposition does not exert a large fitness cost on host plants, sex‐biased interactions can emerge from preferences developed on a hermaphroditic host species, and new nursery pollination interactions can arise as negative or neutral rather than as mutualistic for the plant.

## 1 Introduction

Flowering plants have diverse reproductive strategies. Although most are hermaphroditic, producing flowers that contain both male and female reproductive structures, many angiosperms have adaptations that reduce the likelihood of self‐fertilization. Plants commonly separate female and male sex functions in time (e.g., protandry) and, less commonly, in space (e.g., dioecy, monoecy). In dioecy, the most extreme form of spatial sex separation, individual plants produce only female or only male flowers, making self‐fertilization impossible.

One of the most common evolutionary pathways from hermaphroditism to dioecy involves gynodioecy, where female and hermaphrodite individuals coexist, as an intermediate stage (Charlesworth, 1999). For gynodioecy to arise from hermaphroditism, first a mutation causing male sterility must occur in a hermaphroditic population, creating female individuals (Charlesworth, 1999). If females have a large enough seed production advantage over hermaphrodites, they will persist, stabilizing gynodioecy.

The genetics of sex determination affect the conditions that will determine whether females persist among hermaphrodites and what female frequencies will be stable. Sex can be determined by nuclear male sterility alleles or interactions between nuclear and mitochondrial alleles (hereafter “cytonuclear interactions”), where mitochondrial alleles cause male sterility (creating females) but nuclear alleles restore male function to hermaphrodites (Bailey & Delph, 2007; Lewis, 1941; Lloyd, 1976; Saumitou‐Laprade, Cuguen, & Vernet, 1994). When plant sex is under nuclear control, females must produce at least twice as many seeds as hermaphrodites to persist, but when sex determination is cytonuclear, the relative seed production advantage required by females for their persistence is much smaller, and under particular theoretical conditions females producing only six percent more seeds than hermaphrodites can be sufficient to maintain gynodioecy (Charlesworth, 1981; Charlesworth & Charlesworth, 1978). Female reproductive advantage over hermaphrodites is common in gynodioecious species, but the magnitude varies among species as well sometimes varying among populations or with female frequency within single species (Dufay & Billard, 2012). Female advantage can be expressed through sex differences in fruit number, fruit set (fruits/flowers), seed set (seeds/ovules), seeds per fruit, seeds per plant, seed mass or size, and/or germination rate (Dufay & Billard, 2012). Because cytonuclear gynodioecy can be maintained with a small female seed production advantage, if the initial relative advantage of females compared to hermaphrodites is small, then a minor reduction in female fitness due to biotic or abiotic factors could shift relative fitness below the 1:1 ratio needed to maintain the stability of gynodioecy. Thus, depending on the relative fitness of females and hermaphrodites, small fitness shifts due to abiotic or biotic factors could have large evolutionary implications in systems with cytonuclear gynodioecy.

In dioecious and gynodioecious plants, phenotypic differences between the sexes often affect interactions with pollinators and herbivores (Ashman, 2002; Ashman & Stanton, 1991; Barrett & Hough, 2013). For example, pollinators are frequently more attracted (i.e., make more or longer‐lasting visits) to pollen‐bearing plants because of larger flowers or floral displays (e.g., Ashman, 2000; Asikainen & Mutikainen, 2005; Williams, Kuchenreuther, & Drew, 2000). Herbivores also prefer the pollen‐bearing sex. In 17 of 21 dioecious species from 15 families, male plants suffered significantly more herbivory than females (Ågren, Danell, Elmqvist, Ericson, & Hjältén, 1999) and damage was biased toward hermaphrodites, rather than females, across several gynodioecious taxa (Ashman, 2002).

Ashman (2002) has demonstrated theoretically that sex‐biased damage can promote the evolution of gynodioecy and dioecy from hermaphroditism, especially when the tissues consumed are resource sinks (flowers, fruits, and seeds) rather than sources (leaves). Although Ashman (2002) does not distinguish between nuclear and cytonuclear gynodioecy, she considers the effects of sex‐biased damage on seed production, pollen fitness, and hermaphrodite mating system parameters, which could be important in both nuclear and cytonuclear gynodioecy. Because damage to flowers and fruits directly affects plant reproduction, it is likely to have a stronger effect on female and hermaphrodite fitness (both in terms of pollen and seeds) than leaf damage (Ashman, 2002). Because of their direct effects on plant reproduction, nursery pollination interactions (also known as brood pollination), where an insect species pollinates but also lays eggs in flowers and larvae feed on the plant's reproductive tissues, are good candidates for improving our understanding of how sex‐biased interactions affect the relative fitness of females and hermaphrodites and the maintenance of gynodioecy.

In this study, we evaluated sex bias in a recently established nursery pollination interaction between native *Hadena ectypa* (Morrison) moths and their introduced gynodioecious host plant, *Silene vulgaris* (Moench) Garcke. We addressed the following questions:

1. Is there sex bias in oviposition and damage to plants among and within populations?
2. What plant traits are associated with oviposition?
3. How does receiving eggs affect female and hermaphrodite host plant fruit and seed production?

## 2 Methods

### 2.1 Study system

**Figure 1:**
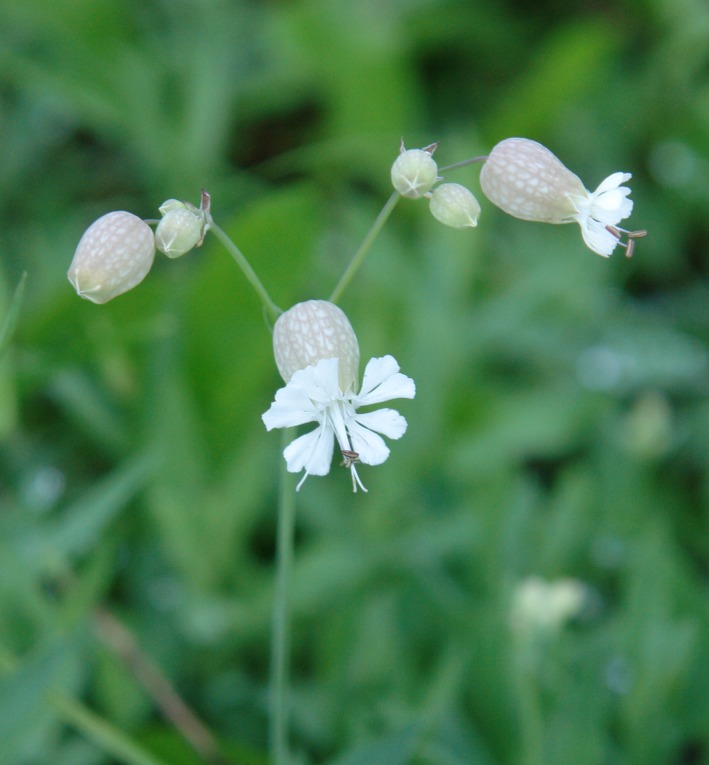
Hermaphrodite *Silene vulgaris* in flower.

Species in the plant genus *Silene* (Caryophyllaceae) engage in diverse nursery pollination interactions, with outcomes ranging from negative to positive with moths from two genera (*Hadena* [Noctuidae] and *Perizoma* [Geometridae]) (Kephart, Reynolds, Rutter, Fenster, & Dudash, 2006). *Hadena* moths can have significant fitness effects on their *Silene* host plants, with *Hadena rivularis* (F.) damaging up to 100% of the available ovules in some European populations of *Silene latifolia* Poir. (Wolfe, 2002). *Hadena ectypa*, a species native to North America, was discovered in western Massachusetts in 2002 (Nelson, 2012). This was the first record of the moth in New England, as its range had previously been thought to stretch no further north or east than southeastern New York state (Nelson, 2012). *Silene stellata* (L.) W. T. Aiton, a hermaphroditic species native to North America, is the known host plant for *H. ectypa* (Nelson, 2012), but *S. stellata* does not occur in Massachusetts (Cullina, Connolly, Sorrie, & Somers, 2011), with the northern edge of its range historically occurring in Connecticut (Nelson, 2012). Since at least 2002, *Hadena ectypa* has been using *Silene vulgaris* (Figure 1) as its host in western Massachusetts (Nelson, 2012). *Silene vulgaris* was introduced from Europe around 200 years ago and is now widely naturalized throughout North America, including in the southeastern US where *S. stellata* also occurs (Nelson, 2012). *Silene vulgaris* is gynodioecious with cytonuclear sex determination (Charlesworth & Laporte, 1998) and has nursery pollination interactions with several *Hadena* moth species in Europe (Pettersson, 1991b).

### 2.2 Sampling

To assess sex bias in *H. ectypa* oviposition on *S. vulgaris*, we surveyed six natural populations in 2014 (Table S1), examining all of the flowers on one *S. vulgaris* stem every 5 m along a transect at each site. We examined single flowering stems because individual plants can have hundreds of stems and plants grew densely at our study sites, making it difficult to identify which stems belonged to particular individuals. Transects traversed populations and ranged from 100 to 600 m in length. At each point along the transect, we examined the nearest stem bearing an open flower. For each stem, we recorded the sex of the flowers (female or hermaphroditic), the number of open flowers, and the number of *H. ectypa* eggs and caterpillars present. Late‐instar *H. ectypa* caterpillars have a distinctive dorsal chevron pattern (Nelson, 2012) that allows them to be discerned from other species likely to occur in most of our study areas (M. W. Nelson, personal communication). As *Hadena capsularis* Guenée is known to occur in Vermont (M. W. Nelson, personal communication), it is possible that either or both *H. capsularis* or *H. ectypa* eggs and caterpillars were observed in our Vermont populations (VBE and VBR). Because we were simply interested in whether oviposition and different forms of damage were sex‐biased in our multipopulation surveys, rather than the effects or preferences of particular interacting species, the potential presence of *H. capsularis* in our Vermont populations does not affect our interpretation of the multipopulation surveys. We also recorded whether each stem had leaf or flower damage, although for this damage we did not know herbivore or florivore identity.

To assess whether oviposition was associated with plant traits other than sex, we focused on our largest *S. vulgaris* population (MSH) in western Massachusetts in 2015 and monitored 80 females and 80 hermaphrodites across the flowering season, using whole plants rather than single stems. We chose these focal plants haphazardly based on having at least one open flower at the time of selection (22 June–6 July 2015). We checked each plant for eggs and late‐instar caterpillars four times over the flowering season (June 22 – July 6, July 20 – 22, July 31 – August 6, and August 17 – 19) and measured plant and floral traits that might influence oviposition (Kula, Dudash, & Fenster, 2013 and references therein): number of open flowers, plant size (projected area, number of stems, and height of tallest flower), and flower size (floral face width and flower depth; Figure S1). Projected area was calculated by multiplying plant length and width obtained by measuring the plant from above along its longest axis for length and at 90 degrees from the length axis for width. For plant‐level floral traits, we averaged the mean of the measurements from two flowers to obtain mean floral trait measurements for each plant. We also assessed damage to floral tissues at the first and third census dates by examining plants for bud, calyx, petal, and ovary damage.

To assess the effect of within‐plant floral variation on oviposition decisions, we collected detailed measurements of floral traits for age‐matched pairs of flowers on individual plants where one flower received an egg but the other did not at MSH in 2015. *Silene vulgaris* flowers progress through predictable stages of sex expression and maturity (Jolls, Chenier, & Hatley, 1994), so we used sex expression to assess the developmental stage of flowers. We assessed the egg‐receiving flower's developmental stage and chose another flower on the same plant that most closely matched this stage, but contained no eggs, as the non‐egg‐receiving flower. We measured the width of the floral face, flower length, calyx width, calyx length, and the diameter of the floral tube opening (Figure S1) for the pairs of egg‐receiving and non‐egg‐receiving flowers. A single observer made all of the measurements and each measurement was made twice. We averaged the two measurements to obtain a single measurement for each trait for each flower.

To determine the effect of oviposition on host plant reproduction, we counted the number of expanded and damaged fruits on each focal plant at the third census date. We counted fruits and assessed the number of predated fruits at this time because it appeared that most plants had finished flowering for the season. We observed new eggs on plants after the fruit count, but did not include these oviposition events in our analyses of traits affecting fruit production and predation. We also counted the number of seeds produced by the egg‐receiving and non‐egg‐receiving flower pairs described above. To assess whether egg‐receiving flowers produced more seeds than non‐egg‐receiving flowers, indicating that they were pollinated effectively, we also counted seeds produced by 10 additional flower pairs at MSH in 2016 from which we removed the egg from the egg‐receiving flower and performed a sham egg removal from the non‐egg‐receiving flower. We removed the eggs from these egg‐receiving flowers because developing larvae would consume fruits and seeds, precluding comparison of seed production. A single observer counted all the seeds.

### 2.3 Statistical analyses

We conducted all statistical analyses in R, version 3.3.1 (R Core Team, 2016). Several of our response variables were binary (i.e., whether plants received eggs or damage), for which we report 95% binomial confidence intervals for these response variables along with observed proportions of outcomes. We used the binom package (Dorai‐Raj, 2014) to calculate binomial confidence intervals with the Pearson‐Klopper exact method. Error bars for figures with binomial response variables are not equal in length above and below the observed proportion because binomial confidence intervals are not symmetric.

#### 2.3.1 Sex‐biased oviposition and damage

In testing for sex bias in oviposition and damage, our null hypothesis was that females and hermaphrodites would receive eggs or damage in proportion to the population sex ratio (at the individual, stem, or flower level, depending on the analysis). For example, in a population that was 10% female and 90% hermaphrodite with no sex bias, we would expect females to receive 10% of the eggs and hermaphrodites to receive 90% of the eggs. If oviposition were female biased, we would expect females to receive significantly more than 10% of the eggs and if oviposition were hermaphrodite biased, we would expect females to receive significantly <10% of the eggs.

We used binomial generalized linear models (GLMs) to test for sex bias in oviposition and damage. The sex term in the model estimates the likelihood of a female or a hermaphrodite receiving an egg. If the sex term is significant, it indicates that one sex is receiving eggs or damage significantly more often than expected based on the underlying sex ratio in the sample. For all GLMs, we used likelihood ratio (LR) tests to assess the significance of the sex term and other predictors of interest by comparing two GLMs that only differed in the presence of the predictor of interest. For our 2014 surveys, we used binomial GLMs to test the effect of plant sex, population, and a sex by population interaction on oviposition. For our 2015 monitoring study, we tested for sex bias in the likelihood of a plant ever receiving an egg using the same binomial GLM approach, with sex as the only predictor.

Because sex at the flower level, rather than the stem or plant level, could be more important to ovipositing insects, we also assessed whether oviposition was sex biased at the flower level for the 2014 multipopulation dataset. We used both binomial glms (as above) and a permutation test for the flower‐level analyses. To conduct the permutation test, we reshuffled whether each flower received an egg among all of the flowers within each population 10,000 times, calculated the number of hermaphrodites that had received eggs for each of those randomizations, and compared the actual number of hermaphrodite flowers that had received eggs to the distribution of simulated hermaphrodite egg receipt. We calculated the permutation *p‐*value (two‐tailed) as twice the number of simulated values that were more extreme than the observed value. We were unable to assess flower level sex bias in oviposition at MSH in 2015 because of our study design: we checked plants for eggs four times throughout the growing season, but only obtained a single flower count for each plant, and 20 of the 47 plants that received eggs did not have any open flowers at the time of the flower count.

#### 2.3.2 Traits associated with oviposition

We used a binomial GLM to assess whether particular plant traits were associated with oviposition. We used all measured plant traits and plant sex as predictors. If plant sex were significant along with other plant traits, it would indicate that sexual dimorphism in unmeasured traits was involved in the observed sex bias. If sex were not significant, but other plant traits were, it would indicate that sexual dimorphism in the measured traits explained any observed sex bias. We tested the significance of each predictor using LR tests and took a backward regression approach to model selection, removing predictor terms from the model one by one until we were left with a model including only the significant predictor variables.

We used paired t‐tests to assess differences in traits and seed production in age‐matched pairs of flowers on plants collected in 2015 where one flower received an egg and the other did not. For 10 additional age‐matched pairs of flowers from 2016, we performed permutation tests, where we reshuffled the number of seeds produced randomly within each pair 10,000 times and took the differences between egg‐receiving flowers and controls each time to obtain a distribution of differences against which to compare the difference between egg‐receiving and control flowers that we actually observed. Our observed difference would be significantly different from 0 if <5% of the randomized differences were more extreme than the observed difference. We performed permutation tests on number of seeds produced and fruit mass because of the small sample sizes.

#### 2.3.3 Flower and leaf damage

We used binomial GLMs to test for sex bias in flower and leaf damage across populations where we observed *H. ectypa* eggs in 2014 and in bud, calyx, petal, and ovary damage in the MSH population twice in 2015.

## 3 Results

### 3.1 *Hadena ectypa* oviposition

**Figure 2:**
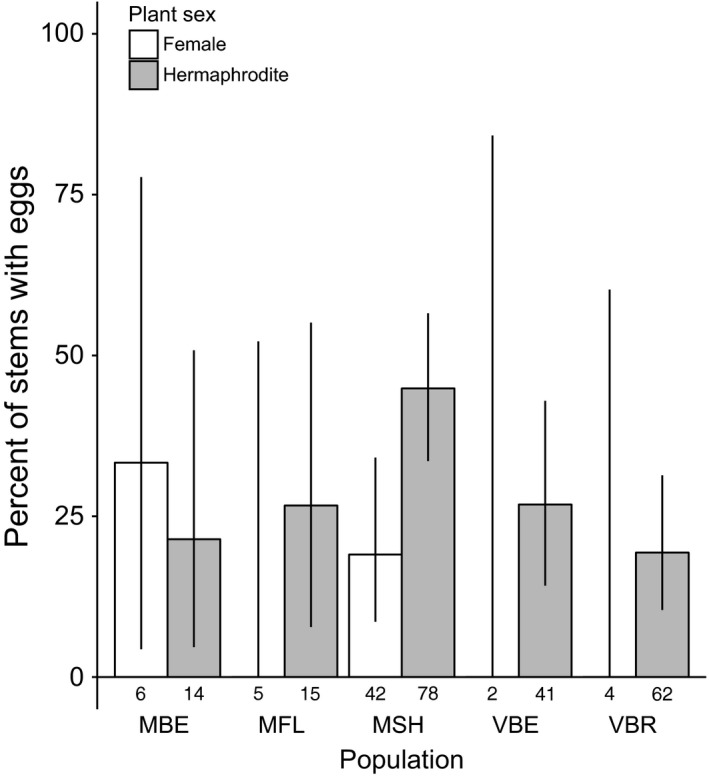
Hermaphrodite *Silene vulgaris* were significantly more likely to receive *Hadena ectypa* eggs than females across populations in 2014. Bars represent observed proportion of female or hermaphrodite stems that received eggs in each population, letters are population codes, and numbers beneath the bars are sample sizes. Error bars are 95% binomial confidence intervals.

**Figure 3:**
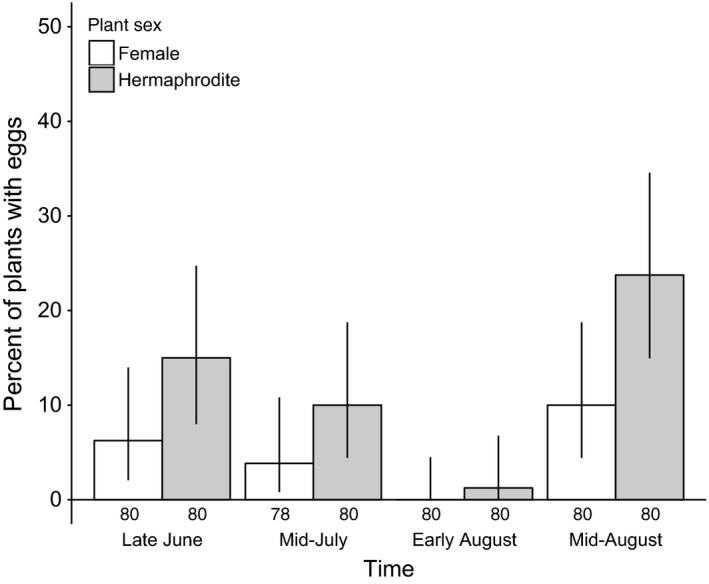
Hermaphrodite *Silene vulgaris* were significantly more likely to receive *Hadena ectypa* eggs than females in 2015 at population MSH. Bars represent observed proportion of female or hermaphrodite plants that received eggs at the time of each census and numbers beneath the bars are sample sizes. Error bars are 95% binomial confidence intervals.

We found eggs and caterpillars in five of the six populations in 2014, with eggs on 18–36% of stems surveyed (Table S1). Caterpillars were quite rare (Table S1), so we did not assess plant traits associated with their presence. In 2014, oviposition was hermaphrodite biased at both stem (*LR* $\chi_{1}^{2}$=* *9.72, *p *=* *.0018; Figure 2) and flower levels (*LR* $\chi_{1}^{2}$ =* *4.90, *p *=* *.027, randomization test *p = *.016) and oviposition frequency varied among populations (*LR* $\chi_{4}^{2}$ =* *12.70, *p *=* *.013; Figure 2), but there was no interaction between plant sex and population (*LR* $\chi_{4}^{2}$ =* *4.46, *p *=* *.35). Oviposition was also hermaphrodite biased at the plant level in the MSH population in 2015 (*LR* $\chi_{1}^{2}$ =* *6.87, *p *=* *.0088; Figure 3). However, when plants received eggs, there was no difference between the sexes in the number of eggs received in either year (2014: *LR* $\chi_{1}^{2}$ =* *1.38, *p *=* *.24; 2015: *LR* $\chi_{1}^{2}$ =* *1.26, *p *=* *.26), probably because moths usually deposited only one egg per stem (2014) or plant (2015) at a time (percent of observations with only one egg at a time: 71% in 2014 and 73% in 2015).

**Figure 4:**
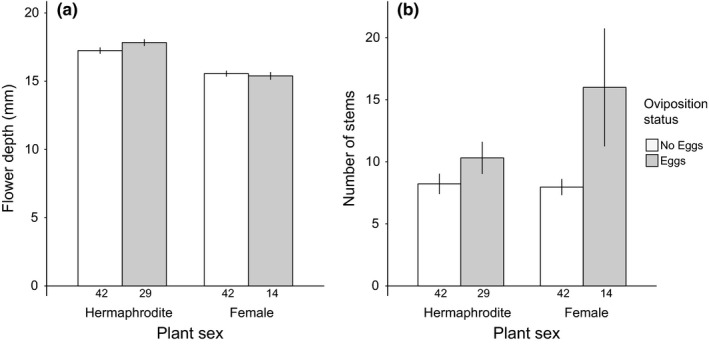
*Silene vulgaris* plants that received *Hadena ectypa* eggs at site MSH in 2015 had deeper flowers (a) and more stems (b) than plants that did not receive eggs. Hermaphrodite *S. vulgaris* plants had deeper flowers than females (a), but there was no sex difference in stem number (b). Numbers beneath bars are sample sizes. Error bars are standard error of the mean.

Some plant traits were associated with oviposition. In the 2015 study, plants with more stems (*LR* $\chi_{1}^{2}$ =* *5.61, *p *=* *.018) and deeper flowers (*LR* $\chi_{1}^{2}$ =* *4.61, *p *=* *.032) were more likely to receive eggs (Figure 4), but height, projected area, number of open flowers, flower width, and sex did not predict oviposition (Table S2). Within a plant, calyx width was the only measured trait that differed significantly between egg‐receiving and non‐egg‐receiving flowers (*t*_35_ **=* *3.15, *p *=* *.0033), with egg‐receiving flowers having wider calyces (mean ± 1*SE*: 8.16 ± 0.22 mm) than non‐egg‐receiving flowers (7.66 ± 0.21 mm) (Figure S2).

Hermaphrodites had significantly deeper flowers than females (*LR F*_1,126_ **=* *60.76, *p *<* *.0001; Figure 4a), but there was no difference between the sexes in number of stems (*LR F*_1,126_ **=* *1.11, *p *=* *.29; Figure 4b). Sexual differences in calyx width could also potentially explain the hermaphrodite‐biased oviposition we observed. Because we only have calyx width measurements for egg‐receiving plants from MSH in 2015, we were unable to directly assess the effect of sexual dimorphism in calyx width on oviposition among plants. However, we tested whether sexual dimorphism existed in calyx width among the egg‐receiving plants on which we tracked the outcome of egg‐receiving and non‐egg‐receiving flowers at MSH, and among 22 females and 18 hermaphrodites grown in a greenhouse from MSH‐collected seed (see Appendix S1 for methods details). There was no sexual dimorphism in calyx width among either of these groups of plants (egg‐receiving: *LR F*_1,34_ **=* *1.28, *p *=* *.27; greenhouse‐grown: *LR F*_1,38_ **=* *0.070, *p *=* *.79).

### 3.2 Consequences of oviposition

#### 3.2.1 Plant level

For the plants monitored at MSH in 2015, number of stems, height, number of flowers present at time of fruit count, and average flower depth predicted fruit production, but flower width, plant area, plant sex, and oviposition status did not (Table 1). Plants that received eggs at MSH in 2015 lost significantly more fruits to apparent *H. ectypa* caterpillar predation than plants that never received eggs (*LR F*_1,133_ **=* *5.36, *p *=* *.022) indicating a fitness cost associated with oviposition. There was also a significant effect of plant sex on fruit loss when oviposition status was accounted for (*LR F*_1,133_ **=* *6.58, *p *=* *.011), such that females lost more fruits than hermaphrodites. However, the sex effect was no longer significant (*LR F*_1,132_ **=* *1.57, *p *=* *.21) when a single extreme fruit loss value was excluded from the analysis, while the oviposition effect remained significant (*LR F*_1,132_ **=* *6.75, *p *=* *.010). The number of fruits lost was relatively small (mean ± 1*SE*: 3.85 ± 0.92 fruits for egg‐receiving plants vs. 1.84 ± 0.32 for non‐egg‐receiving plants) compared to the total number of fruits plants produced (mean ± 1*SE*: 30.75 ± 3.03). Thus, the number of fruits lost to predation was apparently insufficient to affect total fruit production.

**Table 1:** Plant traits associated with *Silene vulgaris* fruit production from Poisson GLM using quasi‐likelihood. Nonsignificant traits were removed one by one from the model to arrive at a final model containing only traits that were significant predictors of fruit production. After the final model was determined, a test statistic (LR F) and *p*‐value for each nonsignificant predictor was obtained by comparing the final model (with all of the significant predictors) to a model containing the significant predictors and the nonsignificant term of interest; these values are reported in the table below for nonsignificant terms. A one‐unit increase in the value of a predictor corresponds to multiplying the response (number of fruits) by the exponentiated coefficient value for that predictor. Degrees of freedom = 1, 120 for each predictor

Predictor	Coefficient	Exponentiated coefficient	Likelihood ratio *F*	*p*
Flower number	0.029	1.029	36.08	<.0001
Stem number	0.020	1.020	17.65	<.0001
Height	0.029	1.029	18.66	<.0001
Flower depth	−0.14	0.87	14.62	.00021
Plant area	0.00011	1.00011	4.74	.031
Flower width	−0.036	0.96	2.71	.10
Plant sex	−0.099 (if hermaphrodite)	0.91	0.39	.53
Oviposition status	−0.10 (if received eggs)	0.90	0.32	.57

#### 3.2.2 Flower level

In 2015, 61% of egg‐receiving flowers and 39% of non‐egg‐receiving flowers failed to produce seeds. For 29 flower pairs where we were able to collect both flowers, neither flower made any seeds in 28% of the pairs, while both flowers made seeds in 31% of the cases. In pairs where both flowers made seeds, there was no difference in fruit mass (*t*_8_ **=* *0.61, *p *=* *.57) or number of seeds produced (*t*_8_ **=* *0.10, *p *=* *.31). A permutation test showed no significant difference (*p *=* *.16) in the number of seeds produced by 10 additional pairs of egg‐receiving vs. non‐egg‐receiving flowers from which we removed eggs in 2016, although the tendency in our sample was for controls to produce seeds more frequently than egg‐receiving flowers. There was also no difference in fruit mass (*p *=* *.12) between egg‐receiving and non‐egg‐receiving flowers from which eggs had been removed.

For the 2015 flower pairs, when flowers produced seeds, there was no difference between the sexes in how many seeds were produced (non‐egg‐receiving flowers: *t*_18_ **=* *0.76, *p *=* *.46; egg‐receiving flowers: *t*_11_ **=* *0.63, *p *=* *.54; Table S3). There were also no sex differences in the mass of fruits that produced at least one seed (non‐egg‐receiving flowers: *t*_18_ **=* *1.5, *p *=* *.15; egg‐receiving flowers: *t*_11_ **=* *1.46, *p *=* *.17).

### 3.3 Flower and leaf damage

**Figure 5:**
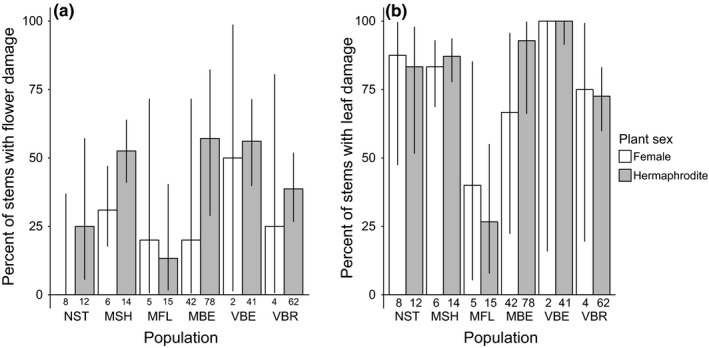
Hermaphrodites were significantly more likely to have flower damage than females (a), but there was no sex bias in leaf damage (b) across populations in 2014. Bars represent the observed proportion of females or hermaphrodites with flower or leaf damage in each population and letters are population codes. The numbers beneath the bars are the number of stems of each sex sampled in each population (Table S1). Error bars are 95% binomial confidence intervals.

Flower damage occurred on 15–56% of stems at populations where we found *H. ectypa* eggs in 2014, while nearly 100% of stems displayed leaf damage (Figure S3), including in the population (NST) without *H. ectypa*. We included all sites surveyed in our analyses of sex‐biased flower and leaf damage and found that hermaphrodites were more likely than females to have flower damage (*LR F*_1,313_ **=* *7.74, *p *=* *.0057; Figure 5a), but there was no sex bias in leaf damage (*LR* $\chi_{1}^{2}$ =* *0.23, *p *=* *.63; Figure 5b). The frequency of both types of damage varied significantly across populations (flower damage: *LR F*_1,317_ **=* *3.24, *p *=* *.0072; leaf damage: *LR* $\chi_{1}^{2}$ =* *46.26, *p *<* *.0001).

**Figure 6:**
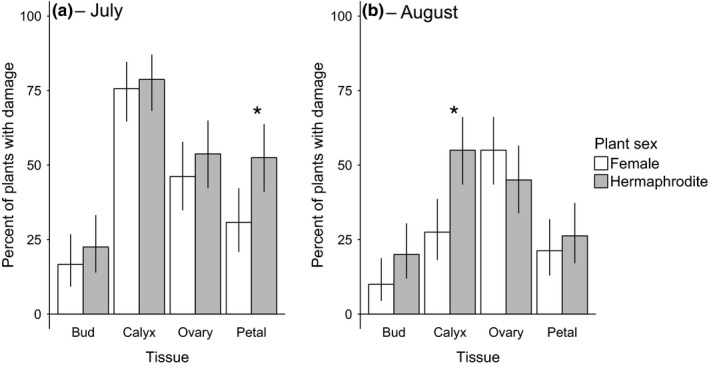
Hermaphrodites at site MSH were more likely than females to have petal damage in July (a) and calyx damage in August (b) 2015, but there was no sex bias in damage to buds or ovaries at either time. Error bars are 95% binomial confidence intervals. Sample sizes: July = 78 females and 80 hermaphrodites; August = 80 females and 80 hermaphrodites. Asterisks indicate significant (*p *<* *.05) differences between females and hermaphrodites.

In 2015, we examined sex bias in bud, calyx, petal, and ovary damage at MSH. Petal damage was hermaphrodite biased in July (*LR* $\chi_{1}^{2}$ =* *7.74, *p *=* *.0054; Figure 6a) and calyx damage was hermaphrodite biased in August (*LR* $\chi_{1}^{2}$ =* *12.67, *p *=* *.00037; Figure 6b). We found no evidence of sex bias in bud or ovary damage at either time (Table S4; Figure 6).

## 4 Discussion

We observed hermaphrodite‐biased oviposition by *H. ectypa* moths on gynodioecious *S. vulgaris* host plants. Flower depth and number of stems predicted oviposition among plants, while within plants, flowers that received eggs had wider calyces than flowers that did not receive eggs. Plant sex was not a significant predictor of oviposition when other plant traits were included in the model, indicating that sex differences in traits included in the model, rather than sexual dimorphism in unmeasured traits, accounted for the observed sex bias in oviposition. Although plants that received eggs lost more fruits to damage, fruit loss was relatively small, resulting in no overall effect of oviposition on total fruit production. There was also no difference in fruit production or the number of seeds per fruit between females and hermaphrodites. Below, we discuss the implications of our results for understanding plant breeding systems, the evolution of mutualism, and moth oviposition preferences.

### 4.1 Sex‐biased interactions and plant breeding systems

Our observations of hermaphrodite‐biased oviposition and flower damage fit the general pattern seen across gynodioecious plant species (Ashman, 2002), but the consequences of hermaphrodite bias for breeding system evolution in our system are not entirely clear. Because females were less likely to receive eggs, we expected them to lose fewer fruits to *H. ectypa* predation than hermaphrodites, but the fruit loss was so minimal that there was no difference in total post‐damage fruit production between the sexes. Plants grew close together at our field site and late‐instar caterpillars are likely to move among plants to find enough young fruits to feed on as they complete development (Nelson, 2012), so it is possible that some oviposition on hermaphrodite hosts led to fruit losses by neighboring female plants.

We found no difference in the number of fruits or seeds per fruit produced by females and hermaphrodites, which was surprising because Taylor, Trimble, and McCauley (1999) found that *S. vulgaris* females produced significantly more fruits than hermaphrodites (but had no difference in flower production) in experimental populations and Olson, Graf, and Niles (2006) found that females produced more seeds per fruit than hermaphrodites in one of two natural North American *S. vulgaris* populations. However, another study (Dulberger & Horovitz, 1984) found no difference in number of seeds per fruit between females and hermaphrodites. Olson et al. (2006) and Taylor et al. (1999) both found that females had higher fruit set than hermaphrodites. We were unable to assess fruit set for our study plants because *S. vulgaris* continuously produces flowers and fruits over a period of several months, and monitoring all flowers and fruits produced was logistically impossible. Another caveat regarding our fruit production data is that because *S. vulgaris* is perennial, there are limitations of a single season of data, especially because there may be sex differences in longevity (Delph, 1999). However, our single‐season data found surprisingly little difference in reproduction between the sexes, suggesting that our population might be close to the critical 1:1 threshold that is important for the maintenance of cytonuclear gynodioecy.

In our system, the ultimate effects of *H. ectypa* on *S. vulgaris* breeding system evolution may also depend on the ecological context. Future work could assess the pollinator and herbivore communities interacting with *S. vulgaris* to determine the relative importance of *H. ectypa* and other non‐ovipositing pollinators and herbivores for female and hermaphrodite host plant fitness. We have observed sweat bees (*Halictidae*), thrips (*Thysanoptera*), earwigs (*Dermaptera*), and ants (*Formicidae*) in *S. vulgaris* flowers during the day (L. A. D. D., personal observation), and we have evidence from a temporal pollinator exclusion experiment that seed production is due to nocturnal, rather than diurnal, pollination (L. A. D. D., unpublished data), but studying pollen donation and removal as well as flower and leaf damage by these different taxa would be helpful. It could also be useful to consider the relative frequencies of flower visits by female (ovipositing) vs. male (non‐ovipositing) *H. ectypa* as well as the frequency of non‐ovipositing visits by female *H. ectypa* moths. However, because oviposition was not associated with increased seed production at the flower level when eggs were removed from flowers, it seems that *H. ectypa*'s role as a pollinator for *S. vulgaris* may be limited.

### 4.2 *Silene–Hadena* interactions and the evolution of mutualism

We found a small fitness cost and no apparent benefits associated with receiving *H. ectypa* eggs, suggesting that the recently established *H. ectypa–S. vulgaris* interaction is mildly negative to neutral. Egg‐receiving plants lost significantly more fruits to predation than plants that did not receive eggs, but did not differ in the total number of expanded fruits. This could be because plants that received eggs were larger and had more flowers than plants that did not receive eggs, mitigating fruit loss, or because *S. vulgaris* plants generally produced large numbers of fruits (>30) and lost small numbers of fruits (<5). For pairs of flowers where one flower received an egg and the other did not, we were surprised by how frequently both flowers failed to expand and set seed (28% of pairs), suggesting a lack of pollination in spite of oviposition by a nursery pollinator. *Hadena ectypa* may be an ineffective pollinator or may oviposit in flowers it has not pollinated, suggesting its relationship in this novel interaction is as more of an antagonist than a mutualist.

Non‐ovipositing co‐pollinators are often present in *Silene–Hadena* and *Silene–Perizoma* nursery pollination systems, often resulting in negative net fitness effects of nursery pollinators (Pettersson, 1991b; Reynolds, Kula, Fenster, & Dudash, 2012; Westerbergh, 2004; Westerbergh & Westerbergh, 2001). For example, in Europe, *S. vulgaris* interacts with several *Hadena* species, including *Hadena bicruris* Hufnagel, *Hadena confusa* Hufnagel, *Hadena perplexa* Denis & Schiffermüller, and *H. rivularis* (Pettersson, 1991b). These *Hadena* species only accounted for 7% of pollen deposition on *S. vulgaris* flowers (Pettersson, 1991b), but consumed 10.6–47.9% of *S. vulgaris* fruits (Pettersson, 1991a), suggesting a strongly negative interaction. *Hadena ectypa*'s interaction with its native host plant, *S. stellata*, is also considered to be negative, as non‐ovipositing co‐pollinators were responsible for the bulk of seed production (Reynolds et al., 2012) and oviposition by *H. ectypa* was associated with flower and fruit destruction (Kula et al., 2013). However, there are also conditions under which the *H. ectypa–S. stellata* interaction may shift toward more positive outcomes for host plants. Reynolds et al. (2012) suggested that the interaction may be mutualistic early in the flowering season and whenever there are high densities of *H. ectypa* moths. Kula et al. (2013) found a link between *H. ectypa* oviposition and *S. stellata* fruit initiation, and that oviposition did not affect the amount of pollen *H. ectypa* delivered to *S. stellata* flowers. Although established *Silene–Hadena* interactions tend to have negative effects on host plant fitness, there are ecological contexts where they can be net positive. Comparing the outcome of the *S. vulgaris–H. ectypa* interaction we describe with these established *Silene–Hadena* systems suggests that nursery pollination interactions can begin as mildly negative to neutral from the host plant's perspective and shift toward strong parasitism or mutualism, depending on ecological context. Of course, the *S. vulgaris*–*H. ectypa* interaction described here represents only one data point, and considering additional recently established interactions would strengthen this conclusion. Plant species that, like *S. vulgaris*, have been introduced to new continents or geographic regions relatively recently provide opportunities to shed light on the evolutionary origins of mutualisms.

### 4.3 Plant traits and oviposition preferences

Female moths should experience selection on oviposition preferences such that they prefer to lay eggs in locations that will maximize survival and growth of their offspring (Castillo, Kula, Fenster, Fenster, & Dudash, 2013). Because of the recent establishment of the *S. vulgaris–H. ectypa* interaction, it is likely that *H. ectypa*'s oviposition preferences on *S. vulgaris* were shaped through interactions with *H. ectypa*'s native host plant, *S. stellata*. On *S. stellata*,* H. ectypa* larvae prefer to feed on young *S. stellata* fruits and adult *H. ectypa* preferentially oviposit in flowers that are young and have not been pollinated (Castillo et al., 2013). *Hadena ectypa* also prefers to deposit eggs in deeper *S. stellata* flowers, on plants with fewer flowers, in larger flowers, and on taller plants (Kula et al., 2013).

We found that *H. ectypa* used both among‐ and within‐plant traits in making oviposition decisions on its new host *S. vulgaris*, some of which correspond to preferences on the native host *S. stellata*. Among *S. vulgaris* plants, flower depth and number of stems affected oviposition. Hermaphrodites had significantly deeper flowers than females, accounting for the hermaphrodite‐biased oviposition we observed. Within plants that received eggs, egg‐receiving flowers had wider calyces than flowers that did not receive eggs. Only flower depth has been consistently associated with *H. ectypa* oviposition on *S. stellata* and on *S. vulgaris*, potentially suggesting that flower depth indicates the extent of floral resources available for adults (nectar) and/or future larvae. Interestingly, the *S. vulgaris* flowers we studied were 6–7 mm deeper on average than *S. stellata* flowers measured by Kula et al. (2013), suggesting that the oviposition preference we observed for deeper flowers was for the deepest available flowers, rather than for *S. vulgaris* flowers that most closely matched preferred phenotypes of the ancestral host plant.

In addition to flower depth, other unmeasured sexually dimorphic qualities might affect oviposition or be correlated with flower depth. Females and hermaphrodites often have chemical differences (nutrient levels, defenses, attractants, and floral rewards) stemming from divergent life‐history strategies (Dawson & Geber, 1999; Eckhart, 1999). *Hadena bicruris* moths use particular floral volatile compounds (lilac aldehydes and phenylacetaldehyde) to locate dioecious *S. latifolia* hosts (Dötterl et al., 2006) and also use smell or taste to differentiate between male and female *S. latifolia* plants (Brantjes, 1976). In *S. vulgaris*, hermaphrodites produce more nectar sugar per flower than females (Jolls et al., 1994). Moths may associate sexually dimorphic traits, like flower depth or floral scent, with higher nectar sugar availability, resulting in the hermaphrodite‐biased oviposition we observed.

## 5 Conclusion

This study adds to the empirical evidence of hermaphrodite‐biased biotic interactions on gynodioecious plant species and identifies plant and flower traits that are associated with hermaphrodite bias. It also highlights that oviposition preferences developed on a hermaphrodite host plant species can lead to sex‐biased oviposition after a shift to a gynodioecious host plant species and shows that both among‐ and within‐plant traits are associated with oviposition. We also show that oviposition did not affect host plant reproduction in terms of fruit number or number of seeds per fruit, suggesting that *H. ectypa* oviposition does not exert a substantial fitness cost on host plants. Further work on this and other *Silene*–*Hadena* nursery pollination interactions could yield a better understanding of the factors that promote the evolution of mutualism vs. parasitism in nursery pollination interactions. Finally, we found no difference between females and hermaphrodites in fruit number or seeds per fruit, suggesting that female and hermaphrodite fitness in our study population may be close to the 1:1 ratio below which cytonuclear gynodioecy would destabilize. Therefore, if biotic interactions cause even small decreases in female fitness, such that female fitness drops below hermaphrodite fitness, these interactions would have the potential to play an important role in shaping future breeding system stability in this system.

## Author Contributions

L. A. D. D. and L. S. A. conceived and designed the study, interpreted the results, and revised the article. L. A. D. D. collected and analyzed the data and drafted the article.

## Conflict of Interest

None declared.

## References

[ece33014-bib-0001] Ågren, J. , Danell, K. , Elmqvist, T. , Ericson, L. , & Hjältén, J. (1999). Sexual dimorphism and biotic interactions In Geber M. A., Dawson T. E., & Delph L. F. (Eds.), Gender and sexual dimorphism in flowering plants (pp. 217–246). Berlin, Heidelberg, and New York: Springer‐Verlag Berlin Heidelberg.

[ece33014-bib-0002] Ashman, T.‐L. (2000). Pollinator selectivity and its implications for the evolution of dioecy and sexual dimorphism. Ecology, 81, 2577–2591.

[ece33014-bib-0003] Ashman, T.‐L. (2002). The role of herbivores in the evolution of separate sexes from hermaphroditism. Ecology, 83, 1175–1184.

[ece33014-bib-0004] Ashman, T.‐L. , & Stanton, M. (1991). Seasonal variation in pollination dynamics of sexually dimorphic *Sidalcea oregana* ssp. *spicata* (Malvaceae). Ecology, 72, 993–1003.

[ece33014-bib-0005] Asikainen, E. , & Mutikainen, P. (2005). Preferences of pollinators and herbivores in gynodioecious *Geranium sylvaticum* . Annals of Botany, 95, 879–886. 15705604 10.1093/aob/mci094 PMC4246743

[ece33014-bib-0006] Bailey, M. F. , & Delph, L. F. (2007). A field guide to models of sex‐ratio evolution in gynodioecious species. Oikos, 116, 1609–1617.

[ece33014-bib-0007] Barrett, S. C. H. , & Hough, J. (2013). Sexual dimorphism in flowering plants. Journal of Experimental Botany, 64, 67–82. 23183260 10.1093/jxb/ers308

[ece33014-bib-0008] Brantjes, N. B. M. (1976). Riddles around the pollination of *Melandrium album* (Mill.) Garcke (Caryophyllaceae) during the oviposition by *Hadena bicruris* Hufn. (Noctuidae, Lepidoptera). I. Proceedings. Series C. Biological and Medical Sciences, 79, 127–141.

[ece33014-bib-0009] Castillo, D. M. , Kula, A. A. R. , Fenster, K. A. D. , Fenster, C. B. , & Dudash, M. R. (2013). Specialist pollinating seed predator exhibits oviposition strategy consistent with optimal oviposition theory. Ecological Entomology, 38, 164–172.

[ece33014-bib-0010] Charlesworth, D. (1981). A further study of the problem of the maintenance of females in gynodioecious species. Heredity, 46, 27–39.

[ece33014-bib-0011] Charlesworth, D. (1999). Theories of the evolution of dioecy In Geber M. A., Dawson T. E., & Delph L. F. (Eds.), Gender and sexual dimorphism in flowering plants (pp. 33–60). Berlin, Heidelberg, and New York: Springer‐Verlag Berlin Heidelberg.

[ece33014-bib-0012] Charlesworth, B. , & Charlesworth, D. (1978). A model for the evolution of dioecy and gynodioecy. American Naturalist, 112, 975–997.

[ece33014-bib-0013] Charlesworth, D. , & Laporte, V. (1998). The male‐sterility polymorphism of *Silene vulgaris*: Analysis of genetic data from two populations and comparison with *Thymus vulgaris* . Genetics, 150, 1267–1282. 9799278 10.1093/genetics/150.3.1267 PMC1460393

[ece33014-bib-0014] Cullina, M. D. , Connolly, B. , Sorrie, B. , & Somers, P. (2011). The vascular plants of Massachusetts: A county checklist. First revision. Westborough, MA, USA: Massachusetts Natural Heritage & Endangered Species Program, Massachusetts Division of Fisheries and Wildlife.

[ece33014-bib-0015] Dawson, T. E. , & Geber, M. A. (1999). Sexual dimorphism in physiology and morphology In Geber M. A., Dawson T. E., & Delph L. F. (Eds.), Gender and sexual dimorphism in flowering plants (pp. 175–215). Berlin, Heidelberg, and New York: Springer‐Verlag Berlin Heidelberg.

[ece33014-bib-0016] Delph, L. F. (1999). Sexual dimorphism in life history In Geber M. A., Dawson T. E., & Delph L. F. (Eds.), Gender and sexual dimorphism in flowering plants (pp. 149–173). Berlin Heidelberg, New York: Springer‐Verlag.

[ece33014-bib-0017] Dorai‐Raj, S. (2014). Binom: Binomial confidence intervals for several parameterizations. R package version 1.1‐1. Retrieved from https://CRAN.R-project.org/package=binom

[ece33014-bib-0018] Dötterl, S. , Jürgens, A. , Seifert, K. , Laube, T. , Weiβbecker, B. , & Schütz, S. (2006). Nursery pollination by a moth in *Silene latifolia*: The role of odours in eliciting antennal and behavioural responses. New Phytologist, 169, 707–718. 16441752 10.1111/j.1469-8137.2005.01509.x

[ece33014-bib-0019] Dufay, M. , & Billard, E. (2012). How much better are females? The occurrence of female advantage, its proximal causes and its variation within and among gynodioecious species. Annals of Botany, 109, 505–519. 21459860 10.1093/aob/mcr062 PMC3278283

[ece33014-bib-0020] Dulberger, R. , & Horovitz, A. (1984). Gender polymorphism in flowers of *Silene vulgaris* (Moench) Garcke (Caryophyllaceae). Botanical Journal of the Linnean Society, 89, 101–117.

[ece33014-bib-0021] Eckhart, V. M. (1999). Sexual dimorphism in flowers and inflorescences In Geber M. A., Dawson T. E., & Delph L. F. (Eds.), Gender and sexual dimorphism in flowering plants (pp. 123–148). Berlin, Heidelberg, and New York: Springer‐Verlag Berlin Heidelberg.

[ece33014-bib-0022] Jolls, C. L. , Chenier, T. C. , & Hatley, C. L. (1994). Spectrophotometric analysis of nectar production in *Silene vulgaris* (Caryophyllaceae). American Journal of Botany, 81, 60–64.

[ece33014-bib-0023] Kephart, S. , Reynolds, R. J. , Rutter, M. T. , Fenster, C. B. , & Dudash, M. R. (2006). Pollination and seed predation by moths on *Silene* and allied Caryophyllaceae: Evaluating a model system to study the evolution of mutualisms. New Phytologist, 169, 667–680. 16441748 10.1111/j.1469-8137.2005.01619.x

[ece33014-bib-0024] Kula, A. A. R. , Dudash, M. R. , & Fenster, C. B. (2013). Choices and consequences of oviposition by a pollinating seed predator, *Hadena ectypa* (Noctuidae), on its host plant, *Silene stellata* (Caryophyllaceae). American Journal of Botany, 100, 1148–1154. 23720431 10.3732/ajb.1200636

[ece33014-bib-0025] Lewis, D. (1941). Male sterility in natural populations of hermaphrodite plants. The equilibrium between females and hermaphrodites to be expected with different types of inheritance. New Phytologist, 40, 56–63.

[ece33014-bib-0026] Lloyd, D. G. (1976). The transmission of genes via pollen and ovules in gynodioecious angiosperms. Theoretical Population Biology, 9, 299–316. 785672 10.1016/0040-5809(76)90050-2

[ece33014-bib-0027] Nelson, M. W. (2012). Notes on a recently discovered population of *Hadena ectypa* (Morrison, 1875) (Noctuidae: Noctuinae: Hadenini) in Massachusetts. The Journal of the Lepidopterists' Society, 66, 1–10.

[ece33014-bib-0028] Olson, M. S. , Graf, A. V. , & Niles, K. R. (2006). Fine scale spatial structuring of sex and mitochondria in *Silene vulgaris* . Journal of Evolutionary Biology, 19, 1190–1201. 16780520 10.1111/j.1420-9101.2006.01103.x

[ece33014-bib-0029] Pettersson, M. W. (1991a). Flower herbivory and seed predation in *Silene vulgaris* (Caryophyllaceae): Effects of pollination and phenology. Holarctic Ecology, 14, 45–50.

[ece33014-bib-0030] Pettersson, M. W. (1991b). Pollination by a guild of fluctuating moth populations: Option for unspecialization in *Silene vulgaris* . Journal of Ecology, 79, 591–604.

[ece33014-bib-0031] R Core Team . (2016). R: A language and environment for statistical computing. Vienna, Austria: R Foundation for Statistical Computing.

[ece33014-bib-0032] Reynolds, R. J. , Kula, A. A. R. , Fenster, C. B. , & Dudash, M. R. (2012). Variable nursery pollinator importance and its effect on plant reproductive success. Oecologia, 168, 439–448. 21833639 10.1007/s00442-011-2095-9

[ece33014-bib-0033] Saumitou‐Laprade, P. , Cuguen, J. , & Vernet, P. (1994). Cytoplasmic male sterility in plants: Molecular evidence and the nucleocytoplasmic conflict. Trends in Ecology & Evolution, 9, 431–435. 21236913 10.1016/0169-5347(94)90126-0

[ece33014-bib-0034] Taylor, D. R. , Trimble, S. , & McCauley, D. E. (1999). Ecological genetics of gynodioecy in *Silene vulgaris*: Relative fitness of females and hermaphrodites during the colonization process. Evolution, 53, 745–751. 28565640 10.1111/j.1558-5646.1999.tb05368.x

[ece33014-bib-0035] Westerbergh, A. (2004). An interaction between a specialized seed predator moth and its dioecious host plant shifting from parasitism to mutualism. Oikos, 105, 564–574.

[ece33014-bib-0036] Westerbergh, A. , & Westerbergh, J. (2001). Interactions between seed predators/pollinators and their host plants: A first step towards mutualism? Oikos, 95, 324–334.

[ece33014-bib-0037] Williams, C. F. , Kuchenreuther, M. A. , & Drew, A. (2000). Floral dimorphism, pollination, and self‐fertilization in gynodioecious *Geranium richardsonii* (Geraniaceae). American Journal of Botany, 87, 661–669. 10811790

[ece33014-bib-0038] Wolfe, L. M. (2002). Why alien invaders succeed: Support for the escape‐from‐enemy hypothesis. American Naturalist, 160, 705–711. 10.1086/343872 18707459

