# Supplementary material for: Sex‐biased oviposition by a nursery pollinator on a gynodioecious host plant: Implications for breeding system evolution and evolution of mutualism
Source: Ecol Evol. 2017 May 23;7(13):4694–703. doi: 10.1002/ece3.3014 (PMC5496538; doi:10.1002/ece3.3014)
Supplement: Supplementary file 1 [file ECE3-7-4694-s001.docx]

Figure S1

Figure S1. Diagram of floral traits measured. Left: side view of *Silene vulgaris* flower showing calyx width, flower depth, and calyx length measurements. Right: front view of *S. vulgaris* flower showing tube opening width and floral face width measurements.

Figure S2

Figure S2. Differences in five floral traits between egg-receiving and control flowers. Egg-receiving flowers had significantly wider calyces than control flowers, but there were no differences between the groups in the other floral traits measured. Sample size = 36 egg-receiving and 36 control flowers. Error bars are ±1 standard error of the mean. Asterisk indicates a significant difference (*P* < 0.05) between egg-receiving and control flowers. See Fig. S1 for details on floral traits measured.

Figure S3

Figure S3. Percent of *S. vulgaris* stems with *H. ectypa* eggs, caterpillars, flower damage, and leaf damage at each of six populations surveyed in 2014. Flower damage was likely due to *H. ectypa* caterpillars, but leaf damage was probably not. Error bars are 95% binomial confidence intervals. Sample sizes: NST = 20; MSH = 120; MFL = 20; MBE = 20; VBE = 43; VBR = 66.

Table S1. Population codes, locations, sex ratios, and *Hadena ectypa* egg and caterpillar abundance at *Silene vulgaris* study sites.

| **Population Code** | **Location** | **Latitude (° N)** | **Longitude (° W)** | **Year(s) Studied** | **Sample Size** | **Sex Ratio  (% Female)** | **# Eggs Observed** | **# Caterpillars Observed** |
| --- | --- | --- | --- | --- | --- | --- | --- | --- |
| NST | Stamford, NY | 42.39360 | 74.60000 | 2014 | 20 | 40 | 0 | 0 |
| MSH | Sheffield, MA | 42.08307 | 73.36524 | 2014, 2015 | 120 (2014); 160 (2015) | 35 (2014) | 71 (2014) | 3 (2014) |
| MFL | Florida, MA | 42.68278 | 73.01811 | 2014 | 20 | 25 | 7 | 3 |
| MBE | Bernardston, MA | 42.68178 | 72.54431 | 2014 | 20 | 30 | 7 | 3 |
| VBE | Bennington, VT | 42.85460 | 72.98096 | 2014 | 43 | 5 | 14 | 5 |
| VBR | Bristol, VT | 44.15323 | 73.04536 | 2014 | 66 | 6 | 14 | 8 |

Table S2. *Silene vulgaris* traits associated with oviposition by *Hadena ectypa* moths. Results are from likelihood ratio tests comparing binomial generalized linear models that differ in the presence of one predictor (trait). Non-significant traits were removed one by one from the model to arrive at a final model containing only traits that were significant predictors of oviposition. After the final model was determined, a test statistic (LR X^2^) and *P*-value for each non-significant predictor was obtained by comparing the final model (with flower depth and stem number as the only predictors) to a model containing the significant predictors and the non-significant term of interest; these values are reported in the table below for non-significant terms. Degrees of freedom = 1 for each test.

| **Trait** | ***LR X^2^*** | ***P*** |
| --- | --- | --- |
| Flower depth | 4.61 | 0.032 |
| Stem number | 5.61 | 0.018 |
| Height | 0.019 | 0.89 |
| Projected area | 0.088 | 0.77 |
| Number of open flowers | 2.38 | 0.12 |
| Flower width | 0.85 | 0.36 |
| Plant sex | 1.22 | 0.27 |

Table S3. Mean seed production by eight pairs of female and 21 pairs of hermaphroditic *Silene vulgaris* flowers at population MSH in 2015. Within a pair, both flowers were on the same individual plant and one flower had received an *H. ectypa* egg (egg-receiving) while the other had not (non-egg-receiving). The non-egg-receiving flower was selected as the flower on the plant that most closely matched the egg-receiving flower’s developmental stage. Only pairs of flowers where both the egg-receiving and non-egg-receiving flowers produced seeds were included in these calculations.

| **Flower Sex** | **Oviposition Status** | **Mean Seeds Produced**  **(± 1SE)** |
| --- | --- | --- |
| Female | Egg-receiving | 18.25 ± 5.99 |
|  | Non-egg-receiving | 20.25 ± 6.05 |
| Hermaphrodite | Egg-receiving | 9.67 ± 3.40 |
|  | Non-egg-receiving | 12.67 ± 3.92 |

Table S4. Results of statistical tests for sex bias in bud, petal, calyx, and ovary damage in July and August 2015 at population MSH. To obtain a *P*-value for each type of damage in each of the time periods, we used likelihood ratio tests to compare binomial generalized linear models that included plant sex as a predictor or included only an intercept. Petal damage was hermaphrodite-biased in July and calyx damage was hermaphrodite-biased in August. Degrees of freedom = 1 for each test.

| **Time** | **Structure Damaged** | ***LR X^2^*** | ***P*** |
| --- | --- | --- | --- |
| July | Bud | 0.86 | 0.36 |
|  | Calyx | 0.22 | 0.64 |
|  | Petal | 7.74 | 0.0054 |
|  | Ovary | 0.91 | 0.34 |
| August | Bud | 3.19 | 0.074 |
|  | Calyx | 12.67 | 0.00037 |
|  | Petal | 0.55 | 0.46 |
|  | Ovary | 1.60 | 0.21 |

Appendix S1

**Methods – *Silene vulgaris* Greenhouse Study**

*Silene vulgaris* plants were grown in a greenhouse at the authors’ institution from seed collected from population MSH in summer 2013. On December 11 and 12, 2013, seeds were planted into black plastic 128-plug trays, with one seed per cell (T.O. Plastics, Clearwater, Minnesota, USA) and maintained in a propagation house with natural light and a constant temperature of 23.89°C until December 21, 2013, when seedlings were transferred to a greenhouse with 14 hours of supplemental light (0600–2200h) and temperatures of 22.22°C during the day and 18.89°C at night. Between December 21, 2013 and January 26, 2013, seedlings were transferred to another greenhouse with 14 hours of supplemental light (0600–2200h) and temperatures of 23.89°C during the day and 18.33°C at night, where they were maintained for the rest of the study. On January 26 and 27, 2014, seedlings were transplanted to individual 164mL Conetainers (model SC10R; Stuewe & Sons, Inc. Tangent, Oregon, USA). The substrate used at all stages was (by volume) 50% High-Porosity Promix^®^ (Premier Tech Horticulture, Quakertown, Pennsylvania, USA), 25% autoclaved topsoil from the University of Massachusetts South Deerfield Farm, and 25% autoclaved washed, screened sand (Home Depot).

A single observer assessed plant sex and measured calyx width on one flower per plant using digital calipers. Calyx width measurements were repeated twice and averaged for each individual plant. We assessed sexual dimorphism in calyx width by performing a likelihood ratio test in R (R Core Team 2016) on two general linearized models predicting calyx width: model one included a plant sex term and an intercept, while model two included only an intercept. We used a Gaussian error structure for both models.

These plants were part of an experiment on the effects of soil nutrients on plant traits, but we found no difference in calyx width between the high and low nutrient treatment groups (LR F_1,40_ = 0.13, *P* = 0.72), nor was there a sex-by-nutrient interaction (LR F_1,38_ = 2.50, *P* = 0.12), so we combined the calyx width measurements for high- and low-nutrient plants in our analysis of sexual dimorphism in calyx width. Plants received either 0.32g (low nutrient treatment) or 1.48g (high nutrient treatment) of Osmocote^®^ 14:14:14 controlled release fertilizer (The ScottsMiracle-Gro Company, Marysville, Ohio, USA) once during the experiment.
